# Effect of sulfurization time on the properties of copper zinc tin sulfide thin films grown by electrochemical deposition

**DOI:** 10.1038/srep32431

**Published:** 2016-09-07

**Authors:** Ali Aldalbahi, E. M. Mkawi, K. Ibrahim, M. A. Farrukh

**Affiliations:** 1 King Abdullah Institute for Nanotechnology, King Saud University, Riyadh 11451, Saudi Arabia; 2 Nano- Optoelectronic Laboratory, Institute of Engineering Research and Materials Technology (IERMT), National Center for Research, Khartoum- Sudan; 3 Nano-Optoelectronics Research and Technology Laboratory, School of Physics Universiti Sains Malaysia, 11800 Penang, Malaysia; 4 Department of Chemistry, GC University Lahore, 54000 Lahore, Pakistan

## Abstract

We report growth of quaternary Cu_2_ ZnSnS_4_ (CZTS) thin films prepared by the electrochemical deposition from salt precursors containing Cu (II), Zn (II) and Sn (IV) metals. The influence of different sulfurization times *t (t* = 75, 90, 105, and 120 min) on the structural, compositional, morphological, and optical properties, as well as on the electrical properties is studied. The films sulfurized 2 hours showed a prominent kesterite phase with a nearly stoichiometric composition. Samples were characterized by X-ray diffraction (XRD), field-emission scanning electron microscopy (FESEM), and Raman and UV-VIS-NIR spectrometer at different stages of work. X-ray diffraction and Raman spectroscopy analyses confirmed the formation of phase-pure CZTS films. (FESEM) shows that compact and dense morphology and enhanced photo-sensitivity. STEM - EDS elemental map of CZTS cross-section confirms homogeneous distribution. From optical study, energy gap was enlarged with a changed sulfurization times in the range of 1.37–1.47 eV.

Kesterite Cu_2_ZnSnS_4_ (CZTS) is a good alternative to current chalcogenide materials, like cadmium telluride and Cu(In,Ga)Se_2_ (CIGS), for use in thin-film solar cells because of the low cost, environmentally friendly, large area deposition and room temperature growth.nontoxic of its constituent elements and abundance. CZTS is suited for use as the absorber layer in photovoltaic devices^1^,^2^ because of its p-type conductivity, high absorption coefficient in the visible solar spectrum (>10^4^ cm^−1^), and direct band gap ranging from 1.4 to 1.7 eV, which is close to the optimum value required for a solar cell absorber layer^3^. The highest efficiency reported for a CZTS-based solar cell under laboratory conditions is 11.1%^4^. The theoretical efficiency of CZTS is about 32.2%^5^, so more studies are required to develop production methods to increase the efficiency of CZTS solar cells. However, it is challenging to grow pure kesterite CZTS without secondary phases such as elemental zinc and tin chalcogenide compounds. Also, the loss of unstable tin metal during sulfurization makes composition and phase control of CZTS difficult. In addition, CZTS absorber films typically contain spurious phases with smaller band gaps than that of CZTS, such as Cu_2_S (1.2 eV), and Cu_2_SnS_3_ (1.0 eV), which can deteriorate the performance of solar cells because they lower the open circuit voltage (V_OC_) and shorten carrier lifetime.

Various methods have been used to prepare CZTS thin films including non-vacuum and vacuum techniques. These methods include radio-frequency magnetron sputtering deposition, screen printing, co-sputtering, photochemical deposition, sol–gel deposition, spray pyrolysis deposition, pulsed laser deposition, thermal evaporation, paste-based methods, and electrodeposition. Unlike vacuum technology and other chemical methods, electrodeposition represents an industrially feasible approach to prepare large-area uniform CZTS precursor films with inexpensive source materials, low energy consumption, and low-cost capital equipment. However, there are some challenges for the electrochemical deposition of CZT film. The standard reduction potentials of Cu, Zn and Sn elements differ widely and hence cause problems for straight forward single step electrodeposition. Sn electrodeposits are often porous, coarse, and non-adherent with formation of whiskers, needles, and dendrites^6^. The electrodeposition of CZT is complicated because electrodeposition of Zn competes with hydrogen evolution reaction (HER) and causes hydrogen embrittlement that hampers growth of smooth electrodeposits^7^.

The aim of the present work is to determine suitable annealing parameters to prepare highly pure CZTS. Two of the most important parameters to prepare CZTS are sulfurization time and annealing temperature. Before annealing, a drying step is carried out at 200–300 °C to maximize solvent evaporation^8^. However, some reports indicate that intermediates like binary sulfide of the constituent elements, bimetallic alloys or ternary Cu_x_SnS_y_ can form at these temperatures^9^. He *et al*.^9^ reported that annealing at 200 °C formed binary alloys, and at 300 °C produced binary/ternary sulfides. Their results reveal that CZTS film properties are strongly influenced by the pre-annealing temperature. However, the effect of sulfurization time on CZTS formation has not yet been reported. In our previous work, we studied influence of substrate temperature on the properties of electrodeposited kesterite CZTS^10^, and the effect of sulfurization temperature on CZTS thin films^11^. The results show that the crystallinity of the samples improved as a result of thermal annealing. The grain size increased with increasing the annealing temperature. The Cu/(Zn + Sn) and the Zn/Sn ratios changed depending on annealing temperature. Generally, the substrate temperature studies revealed that increasing the annealing temperature of the samples led to improvement of the CZTS thin film properties. The influence of annealing time on the properties of CZTS thin films is important because freshly deposited CZTS thin films may contain defects such as voids and pinholes. Annealing of thin films lowers the defect content, increases crystallite size, and induces recrystallization, which can improve the power efficiency of solar cells.

In this work, wurtzite-structured CZTS thin films composed of pure CZTS are synthesized by electrochemical deposition. The influence of sulfurization time on the structural properties, phase purity and photoelectric properties of the wurtzite-structured CZTS thin films are studied in detail for the first time. We also determine the effects of annealing time on the composition, crystallographic structure, and morphology of CZTS thin films.

## Experimental Section

First, copper zinc tin alloy (CZT) thin films were fabricated by electrochemical deposition using a potentiostat/galvanostat (E-corder 401, eDAQ, Australia). Electrodeposition of the CZT layer was performed using a conventional three-electrode system with Ag/AgCl in saturated KCl as the reference electrode and Pt mesh as the counter electrode. Molybdenum-coated soda-lime glass (20 Ω/sq, 20 × 20 mm) was used as the working electrode. Before deposition, the working electrode was cleaned sequentially with isopropanol, methanol, acetone, and distilled water, and then dried in an oven. The working electrode was then immersed in 25 vol.% ammonia solution for 10 min to remove the molybdenum oxide layer on its surface. The electrochemical deposition of CZT was performed potentiostatically using an applied triangular wave pulse from 0 to 1.2 V *vs*. Ag/AgCl.

Electrochemical experiments were performed at room temperature in an aqueous electrolyte containing Cu(OAc)_2_.H_2_O (0.886 g, 4.3 mmol), Zn(OAc)_2_.2H_2_O (0.675 g, 3.0 mmol), and Sn(OAc)_2_.2H_2_O (0.541 g, 2.5 mmol) in deionized water (50 mL) containing concentrated trisodium citrate (5 mL) as a complexing agent. The electrolyte was adjusted to pH = 4 with sodium hydroxide solution and lactic acid before use.

CZT layers were deposited on Mo-coated glass substrates in a single-step electrodeposition process. The deposition time was 1 h and the electrolyte was purged with nitrogen prior to the electrodeposition. After completing the electrodeposition, the samples were rinsed with distilled water and dried in air. After electroplating, the CZT precursor layers were loaded into a graphite container and inserted into the three-zone quartz tube furnace for different times of 75, 90, 105, and 120 min. The tube was filled with nitrogen and the samples were then heated at a rate of 20 °C/min. A total of 20 mg Sulfur was evaporated from pellets (99.999% purity) at 400 °C from a source placed at the left end of the furnace (first zone) and a very low flow rate of argon. The samples were heated for different times at 580 °C in the second heating zone. The samples were then allowed to cool to room temperature naturally. Following annealing, KCN etching was performed by immersing the sulfurized thin films in aqueous KCN solution (0.1 M) for 1 min.

Composition analysis and film morphology were examined by energy-dispersive X-ray (EDS) analysis, which was performed using a field-emission scanning electron microscope (FESEM; Nova Nano SEM 450, FEI, Japan) equipped with an EDS analyzer. Structural characterization of the films was carried out by X-ray diffraction (XRD) in the 2*θ* range from 20° to 80° on an X-ray diffractometer (PANalytical X’pert PRO MRD PW3040, the Netherlands) with Cu K*α* radiation (*λ* = 0.15406 nm). The optical properties of the films were investigated using an ultraviolet–visible–near infrared spectrophotometer (UV–Vis–NIR; Cary 5000-UV BROP, Agilent Technologies, Australia).The electrical properties were characterized by Four probe Hall Effect measurements at RT using the HL5500PC system- Australia).

## Results and Discussion

**Figure 1:**
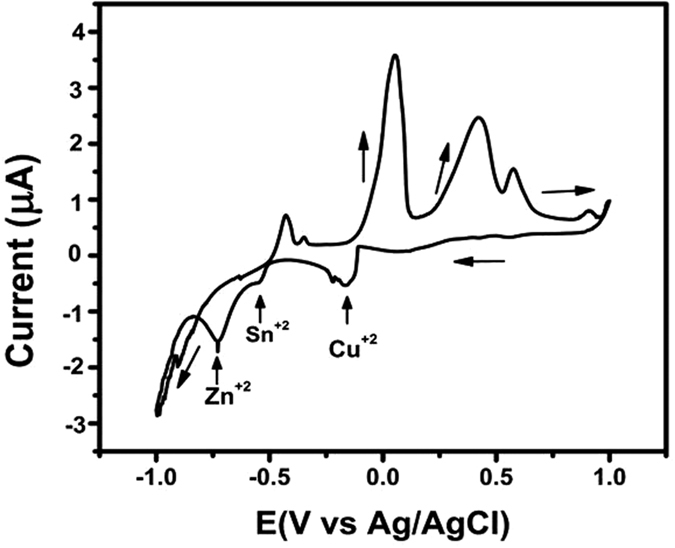
Cyclic voltammogram (scan rate: 10 mV s^−1^) and calculated reduction potentials of copper, zinc and tin from an electrolyte solution containing Cu(OAc)_2_.H_2_O (4.3 mmol), Zn(OAc)_2_.2H_2_O (3.0 mmol), Sn(OAc)_2_.2H_2_O (2.5 mmol), and concentrated tri-sodium citrate (5 mL) at pH = 4.

Voltammograms were recorded in the range from −1 to 1 V (vs. Ag/AgCl) at a scan rate of 10 mV s^−1^ to investigate the growth parameters and optimize the co-electrodeposition potential. The reduction potentials of the Cu, Sn and Zn ions are shown in Fig. 1. Cu^2+^ displays a broad peak around −0.26 V (*vs*. Ag/AgCl). In the case of Sn, a peak is observed at around −0.6 V (*vs*. Ag/AgCl). The potential required to deposit Zn was −0.76 V (*vs*. Ag/AgCl).

**Figure 2:**
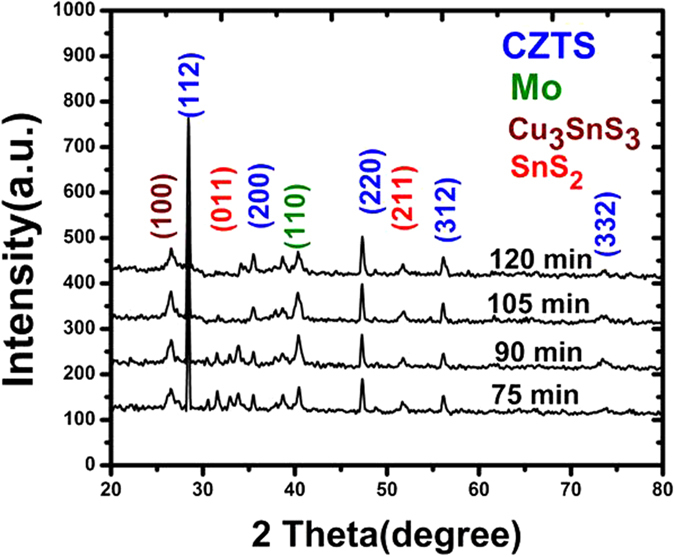
Powder XRD patterns of CZTS thin films obtained after sulfurization times of 75, 90,105, and 120 min.

Powder X-ray diffraction (XRD) patterns obtained for CZTS thin films annealed for different periods are compared in Fig. 2. The samples show four broad peaks that can be correlated with the four main peaks in the powder diffraction file of bulk kesterite CZTS (JCPDS 26-0575, *a* = 5.435 Å, *c* = 10.869 Å). The reflections could also be indexed as 112, 200, 220, 312 and 332. These results are in agreement with those reported by other groups^12^,^13^. However, the XRD patterns of the samples obtained after sulfurization times of 75 and 90 min show more other peaks than those of the samples obtained after sulfurization times of 105 and 120 min. The extra peaks are attributed to the presence of secondary phases of SnS_2_ at 31.53° (011) (JCPDS 14-620), and Cu_3_SnS_3_ at 26.46° (100) (JCPDS 29-0584). A signal from the Mo back contact appeared at 40.5° (110) (JCPDS 01-089-4896). The intensity of the diffraction peaks increased gradually as the annealing time lengthened, indicating the improvement of the crystallinity of the samples. The intensity of the peak from the (112) preferred orientation of CZTS after annealing for 120 min was stronger than those of CZTS thin films prepared using shorter annealing times. This means that the CZTS thin films with the highest crystallinity were obtained after annealing for 120 min. Using the Scherrer’s formula, we calculated the crystallite size in the samples from the XRD data:


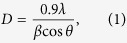


where *D* is the average crystallite size (diameter), *λ* = 0.15406 nm, *B* is the full width at half-maximum (FWHM, rad), and *θ* is the Bragg angle (°). Using Eq. 1, the estimated crystallite sizes of the samples annealed for 75, 90, 105, and 120 min are about 33, 43, 49, and 55 nm, respectively.

**Figure 3:**
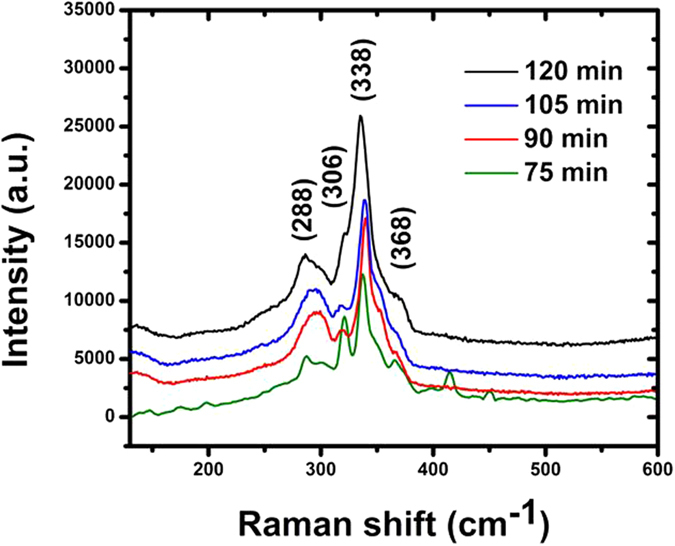
Raman spectra of CZTS thin films obtained after sulfurization times of 75, 90, 105, and 120 min.

In addition to the different structures of CZTS, secondary phases such as ZnS (JCPDS 01-0792 and 05-0566), Cu_2_S, and Cu_3_SnS_4_ (JCPDS 33-0501) are also likely to exist in the films. This is because CZTS is only thermodynamically stable in a narrow region according to the phase diagram. The existence of these secondary phases can greatly influence the performance of devices because these secondary phases can induce differences in the band alignment or a mismatch in the crystal structure. Raman spectroscopy was used to study the composition of the CZTS thin films annealed for different times. To confirm the phase purity or detect the presence of other possible secondary phases, Raman spectroscopy of the samples was conducted over the range 130–500 cm^−1^; the results are presented in Fig. 3. The formation of CZTS was confirmed by the presence of an intense peak at 338 cm^−1^ and weak peaks at 288, 306, and 368 cm^−1^, which are characteristic of CZTS and close to reported values^14^. These four main peaks were perfectly matched with the main Raman peaks of kesterite CZTS^15^. Additionally, the high intensity of the major peak at 338 cm^−1^ in all the Raman spectra indicates the high crystalline quality of our films. Importantly, the Raman spectra did not contain any characteristic peaks of impurities such as Cu_2-x_S (475 cm^−1^), ZnS (351 and 274 cm^−1^), and Cu_3_SnS_4_ (318, 348, and 295 cm^−1^).

**Figure 4:**
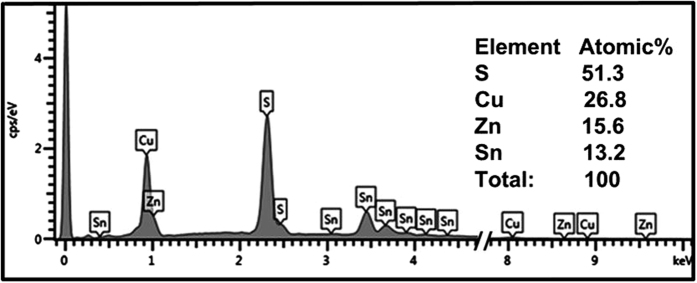
EDX spectrum of the CZTS thin film sulfurized at a substrate temperature of 580 °C for 120 min.

Stoichiometry and crystalline quality of the absorber material play important roles in solar cell performance. Therefore, it is very important to understand and optimize the growth and phase formation of photovoltaic materials to achieve the desired stoichiometry and optimal crystallinity. Figure 4 and Table 1 show the elemental compositions of the synthesized CZTS thin films annealed for different times using the energy-dispersive X-ray (EDX) spectroscopy. The compositions of the synthesized CZTS thin films changed only slightly with annealing time. The theoretical stoichiometry of CTZS is 2 Cu:1 Zn:1 Sn:4 S. The elemental compositions of all our samples were similar to the theoretical ratio.

Table 1 shows the average Cu/(Zn + Sn) and (Zn/Sn) ratios of the CZTS thin films annealed for different times determined using EDX spectroscopy. The sample annealed for 75 min possessed average Cu/(Zn + Sn) and (Zn/Sn) composition ratios of 1.05 and 0.84, respectively. These values are quite close to the stoichiometry of CZTS, but slightly Cu rich and Zn poor. The sample annealed for 90 min had Cu/(Zn + Sn) and (Zn/Sn) ratios of 1.12 and 1, so is rich in both Cu and Zn. When the annealing time was 105 min, the composition of CZTS was almost stoichiometric, although slightly Cu rich and Zn poor. An annealing time of 120 min caused the Cu/(Zn + Sn) ratio to decrease to 0.93. Therefore, an annealing time of 120 min was necessary to obtain CZTS thin films with the desired properties.

The chemical composition of the thin films could be controlled by changing the molar fractions of Cu, Zn, and Sn ions in the preparation. It has been reported for bulk thin-film solar cells that Cu-poor and Zn-rich CZTS light-absorbing layers exhibited higher photoactivity than that of light-absorbing layers with stoichiometric composition^16^. This is because the Cu-poor composition enhances the formation of Cu vacancies, which behave as shallow acceptors in CZTS, while a Zn-rich composition suppresses the substitution of Cu in Zn sites, which results in relatively deep acceptors^17^. Additionally, this ratio necessary to minimize the number of defect clusters [Cu_Zn_ + Sn_Zn_] and [2Cu_Zn_ + Sn_Zn_], which adversely affect the performance of kesterite solar cells.

**Figure 5:**
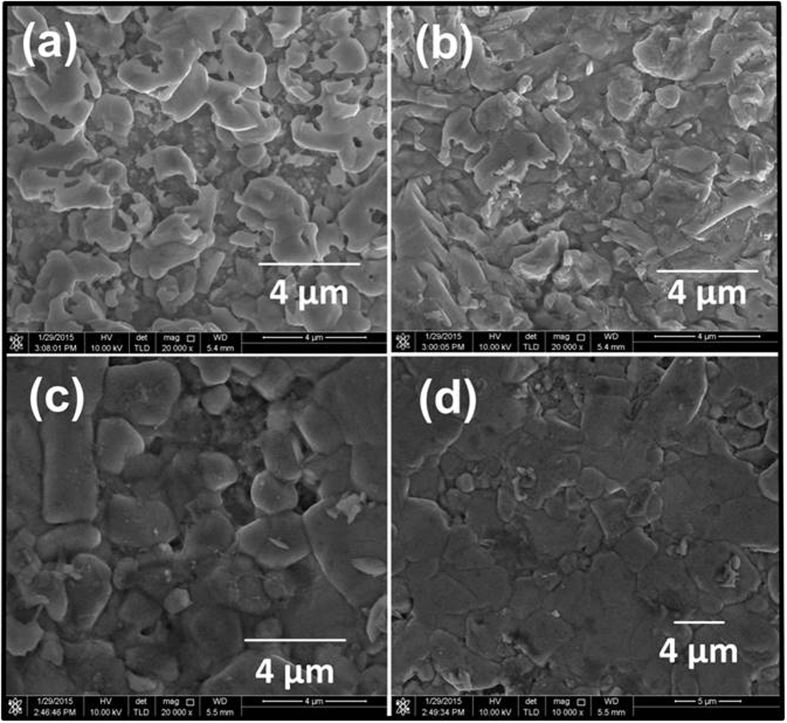
FE-SEM images of CZTS thin films annealed for (**a**) 75, (**b**) 90, (**c**) 105 and (**d**) 120 min.

Field-emission scanning electron microscopy (FESEM) images of the surfaces of CZTS films annealed for different periods are shown in Fig. 5. The morphology of the CZTS films depends on annealing time. The film annealed for 75 min (Fig. 5a) shows the non-uniform texture, with some voids or cavities. Meanwhile, annealing for 90 min produced a film with more apparent grain structures and homogeneous surface morphology (Fig. 5b). As the annealing time increased up to 120 min, the film surface became more compact with large densely packed grains. The FESEM images reveal that the surface morphology of CZTS films strongly depends on the annealing time.

One possible explanation for the observed morphological improvement with lengthening annealing time is the liquid-assisted grain growth, similar to that observed for CIGS^18^. The relatively flat film surface shown in Fig. 5d also indicates the possibility of liquid-assisted grain growth^19^. Furthermore, the large morphological differences between the samples in Fig. 5(b,c) might be caused by the quantities of the liquid phase depending on the deposition time. The FESEM image of the CZTS sample annealed for 120 min (Fig. 5d) indicates that the CZTS thin film contains large grains with a size of 4 μm; this film also exhibits dense and void-free morphology. The large grains in this film can be beneficial to the V_OC_ of a CZTS device because the recombination of photogenerated carriers at grain boundaries can be lowered. The morphology of this CZTS layer featuring a high density of stacking faults along the back contact should contribute to increased lifetime, low recombination losses, and decreased series resistance in devices.

**Figure 6:**
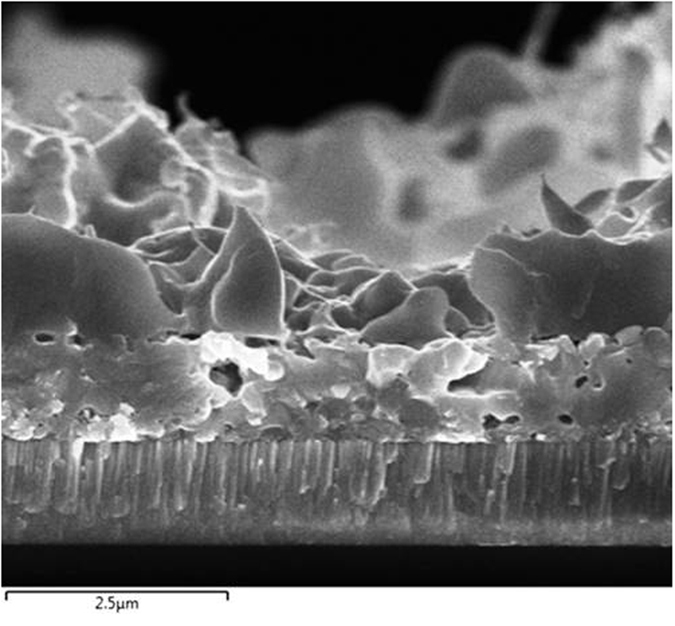
Cross-sectional FESEM image of a CZTS film after sulfurization at 580 °C for 120 min.

Figure 6 displays a cross-sectional FESEM image of a CZTS film sulfurized at 580 °C for 120 min. This CZTS film is almost pinhole free after sulfurization for 120 min, and the film thickness (~2.4 μm) increases compared with that of the as-deposited film (~1 μm). The large grains in the CZTS film indicate that it is highly crystalline, which is consistent with the strong CZTS diffraction peak in the corresponding XRD pattern.

**Figure 7:**
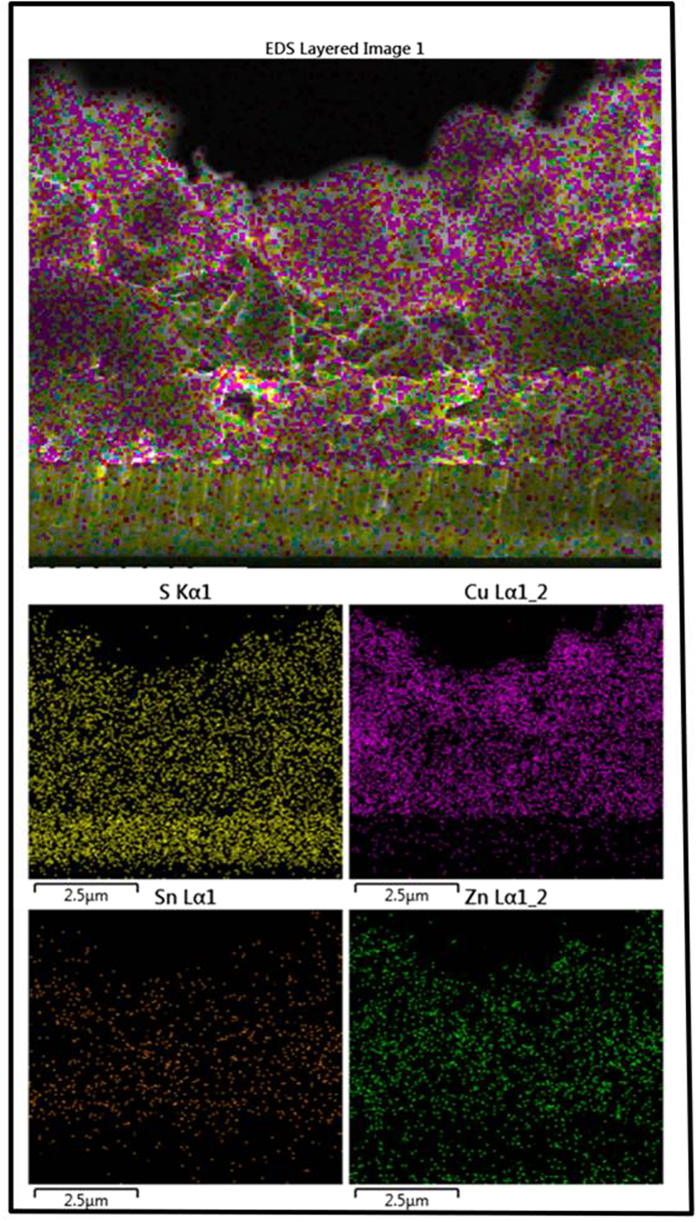
STEM–EDS elemental maps of the cross-section of a CZTS thin film after sulfurization at 580 °C for 120 min.

To confirm the sample composition, and verify that all four elements were present in the samples, scanning transmission electron microscopy (STEM)–EDS elemental maps of a cross section of a CZTS thin film sulfurized at 580 °C for 120 min were obtained (Fig. 7). The STEM–EDS analysis indicates that the four elements Cu, Sn, Zn, and S are homogeneously distributed within the film. The uniform distribution of elements is benefit to the adhesion of the final CZTS layer on the Mo substrate. The huge non uniform distribution will introduce stress in the film inevitable, which was deteriorating for the adhesion of the CZTS layer and the Mo substrate^20^,^21^.

**Figure 8:**
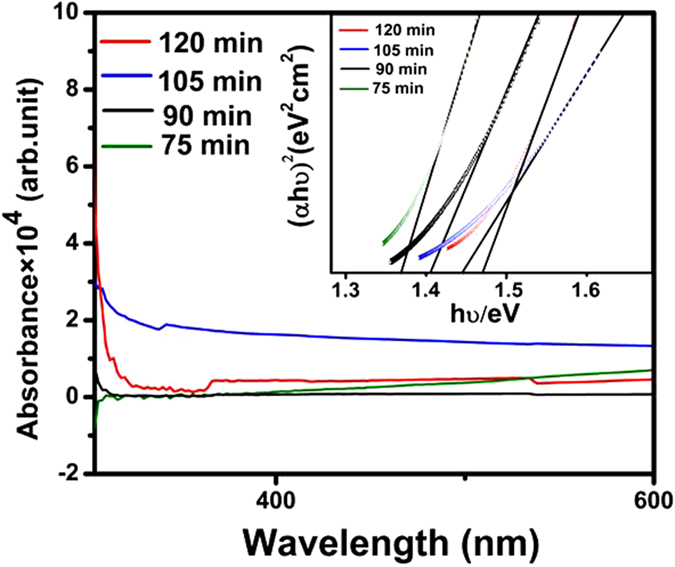
UV-vis absorption spectra of CZTS thin films annealed for different times. The optical band gaps of the CZTS thin films are plotted in the inset.

Near the absorption edge or in the strong absorption zone of the transmittance spectra of materials, the absorption coefficient is related to the optical energy gap, *E*_*g*_, which can be determined by the Tauc’s formula:


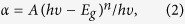


where *A* is an energy-independent constant, *h* is the Planck constant, ν is frequency, and *n* is an index that characterizes the optical absorption process and is theoretically equal to 2 and ½ for indirect and direct allowed transitions, respectively. The absorbance spectra of the CZTS thin films were recorded and are displayed in Fig. 8. All the CZTS thin film samples exhibited broad absorption in the visible region. The absorption coefficients of the sample annealed for 120 min exceeded 104 cm^−1^ in the visible region, which supports that the deposited CZTS material possessed a direct band gap. Extrapolation of the straight line to zero absorption coefficient (α = 0) allows estimation of *E*_*g*_. That is, the band gaps were obtained by plotting (*αhυ*)^2^ *versus* the energy in eV and extrapolating the linear part of the spectrum (*hυ*). The inset of Fig. 8 shows the band gaps of the CZTS thin films prepared using different sulfurization times. Annealing for 75, 90, 105, and 120 min gave CZTS thin films with *E*_*g*_ of 1.37, 1.41, 1.44, and 1.47 eV, respectively, which are close to the optimum value for solar photoelectric conversion of 1.5 eV^22^,^23^. The differences of the band gaps and absorption spectra of the thin films may be caused by the changing particle size and morphology of the CZTS thin films with annealing times. Although composition dependency of *E*_*g*_ has been observed for multinary semiconductor particles like CZTS and CuInS_2_, there has been little investigation of the influence of the particle composition on light−electricity conversion efficiency, especially for CZTS thin films^24^.

To study the electrical properties of kesterite CZTS thin films, the four-probe method using a Hall-effect measurement system at room temperature was employed. Aluminum spot electrodes were made on the surfaces of the CZTS films by thermal evaporation. Table 2 summarizes the Hall mobility, electrical resistivity and carrier concentration of the kesterite CZTS thin films prepared using different annealing times. When the annealing time was 75 min, the resulting kesterite CZTS film showed a low carrier concentration of 2.88 × 10^18^ cm^3^, high Hall mobility of 8.35 cm^2^/V s, and high resistivity of 70.85 Ω cm. When the annealing time was lengthened to 120 min, the resulting kesterite CZTS film exhibited a high carrier concentration of 5.12 × 10^19^ cm^3^, low Hall mobility of 3.09 cm^2^/V s, and low resistivity of 39.46 Ω cm. These results provide further evidence that CZTS thin film properties are strongly affected by annealing time.

Generally, annealing for an extended period assists the diffusion of each element and leads to the formation of homogeneous films. The CZT alloys react with S at high annealing temperature to directly generate CZTS, which results in the formation of CZTS thin films with lower loss of Sn, higher purity and better crystallinity than those obtained at lower temperature.

## Conclusion

We examined the effects of sulfurization time on the properties of CZTS thin films fabricated by single-step electrochemical deposition followed by sulfurization at 580 °C under argon atmosphere. The effects of sulfurization time on the structural, morphological, electrical, and optical properties of the CZTS films were investigated in detail. Polycrystalline CZTS thin films with kesterite crystal structure were obtained after annealing at 580 °C for 120 min. These films exhibited a quite smooth, dense and uniform topography on Mo-coated glass substrates. These CZTS thin films with a mean crystallite size of 55 nm were characterized by broad XRD responses, which provided inconclusive determination of their phase purity. The films annealed for different periods all showed the distinct diffraction peaks of the single pure phase of CZTS with kesterite structure. EDX analysis revealed that the sample annealed for 120 min was nearly stoichiometric, but slightly Cu deficient and Zn rich in composition, which is suited to solar-cell applications. The surface morphology of CZTS thin films annealed for 120 min was homogeneous and compact with a grain size of about 4 μm. STEM–EDS elemental maps of a cross-section of a CZTS thin film confirmed the homogeneous distribution of elements in it. Meanwhile, UV–Vis absorption spectroscopy revealed that the CZTS thin films annealed for 120 min possessed an energy gap of about 1.47 eV and strong optical absorption exceeding 10^4^ cm^−1^ in the visible region, indicating the suitable optical property of as-prepared CZTS thin film for efficient solar energy conversion. Consequently, all results support the proposition that a simple and low-cost single-step electrochemical deposition method followed by sulfurization at 580 °C for 120 min approach is applicable to CZTS synthesis, and the corresponding products are potentially suitable for solar cell applications.

## Additional Information

**How to cite this article**: Aldalbahi, A. *et al*. Effect of sulfurization time on the properties of copper zinc tin sulfide thin films grown by electrochemical deposition. *Sci. Rep.* **6**, 32431; doi: 10.1038/srep32431 (2016).

## Figures and Tables

**Table 1:** Composition of films deposited using different sulfurization times determined by EDX analysis.

Sulfurization time (min)	Cu (%)	Zn (%)	Sn (%)	S (%)	([Cu]/([Zn] + [Sn])	[Zn]/[Sn]
75	27.4	11.4	13.5	47.5	1.05	0.84
90	27.7	12.4	12.3	48.7	1.12	1.00
105	25.6	14.5	12.9	47.5	1.00	1.12
120	26.8	15.6	13.2	51.3	0.93	1.19

**Table 2:** Electrical properties of CZTS films annealed for different times.

Annealing time (min)	Hole concentration (cm^3^)	Hall mobility (cm^2^ V^−1^ S^−1^)	Resistivity (Ω cm)
75	2.88 × 10^18^	8.35	70.85
90	3.34 × 10^18^	6.66	53.27
105	4.56 × 10^19^	4.46	40.13
120	5.12 × 10^19^	3.09	39.46
